# The prevalence of intimate partner violence and risk factors for women and men in China during the Shanghai 2022 lockdown

**DOI:** 10.1017/S2045796024000155

**Published:** 2024-03-19

**Authors:** Liying Yang, Amy Shaw, Thomas J. Nyman, Brian J. Hall

**Affiliations:** 1 The School of Psychology and Cognitive Science, East China Normal University, Shanghai, China; 2 Center for Global Health Equity, NYU Shanghai, Shanghai, China; 3 Department of Psychology, Faculty of Social Sciences, University of Macau, Taipa, China; 4 Faculty of Arts and Sciences, NYU Shanghai, Shanghai, China

**Keywords:** COVID-19, gender, intimate partner violence, lockdown

## Abstract

**Aims:**

Intimate partner violence (IPV) is a global public health concern with negative effects on individuals and families. The present study investigated the prevalence, risk factors and gender disparities associated with IPV during the Shanghai 2022 Covid-19 lockdown – a public health emergency which may have exacerbated IPV.

**Methods:**

We estimated the total IPV prevalence and prevalence of physical, sexual and verbal IPV by using an adapted version of the Extended-Hurt, Insult, Threaten, Scream scale. This cross-sectional study was carried out using a population quota-based sampling of Shanghai residents across 16 districts during the 2022 Shanghai lockdown (*N* = 2026; 1058 men and 968 women).

**Results:**

We found a distinct gendered dynamic, where women reported a significantly higher prevalence of experienced IPV (27.1%, 95% confidence interval [CI]: 23.1–31.4) compared to men (19.8%, 95% CI: 16.1–24.0). Notably, the prevalence estimate mirrored the national lifetime IPV prevalence for women but was over twice as high for men. In multivariable logistic regression analyses, economic stress (income loss: adjusted OR [aOR] = 2.42, 95% CI: 1.28–4.56; job loss: aOR = 1.73, 95% CI: 1.02–2.92; financial worry much more than usual: aOR = 1.89, 95% CI: 1.00–3.57) and household burden (one child at home: aOR = 1.81, 95% CI: 1.12–2.92; not enough food: aOR = 1.67, 95% CI: 1.04–2.70) were associated with increased odds of overall IPV victimization among women but not men. With regard to more serious forms of IPV, job loss (aOR = 2.27, 95% CI: 1.09–4.69) and household burden (two or more children at home: aOR = 2.95, 95% CI: 1.33–7.69) were associated with increased odds of physical IPV against men. For women, a lack of household supplies was associated with increased odds of physical IPV (water: aOR = 3.33, 95% CI: 1.79–6.25; daily supplies: aOR = 2.27, 95% CI: 1.18–4.35). Lack of daily supplies (aOR = 2.17, 95% CI: 1.03–4.55) and job loss (aOR = 2.66, 95% CI: 1.16–6.12) were also associated with increased odds of sexual IPV.

**Conclusions:**

Although a larger proportion of women reported IPV, men experienced greater IPV during the lockdown than previously estimated before the pandemic. Economic stressors, including job loss, and household burden were critical risk factors for serious forms of IPV. Improving gender equality that my account for disparities in IPV in China is critically needed. Policies that mitigate the impact of economic losses during crises can potentially reduce IPV.

## Introduction

Intimate partner violence (IPV) refers to actual or threatened behaviours by a current or former intimate partner that cause physical, sexual or psychological harm, including acts of physical aggression, sexual/reproductive coercion, and psychological abuse and controlling behaviours (World Health Organization, 2010). The impact of IPV on the health and well-being of individuals and families is often devastating, and IPV is recognized as a global public health problem (Hou *et al.*, 2010; World Health Organization, 2022). In the context of mainland China, studies reported a gender imbalance in experiencing IPV. According to Hu *et al*. (2021), in mainland China, the lifetime prevalence of experiencing IPV victimization is 25% for women and 8% for men. A similar gender imbalance of IPV victimization has also been observed globally (Ansara and Hindin, 2010; Archer, 2002; Gass *et al*., 2021; Heath *et al.*, 2013; Oram *et al.*, 2022) and, moreover, research shows that globally women are overrepresented in terms of severe injuries and deaths due to IPV (World Health Organization, 2010). Nonetheless, both men and women may act as either perpetrators or victims. Available IPV research from Western countries found support for both gender symmetry (mostly concerning yelling, shouting and less severe forms of physical violence; Archer, 2006; Straus, 2011) and asymmetry (mainly involving severe aggression such as kicking, choking, restricting physical freedom or sexual violence; Archer, 2006; Hamberger and Larsen, 2015). Therefore, in examining the prevalence of gender-based IPV and its associated factors, the nature or types of violence and sample characteristics need to be taken into account.

Complicating the matter further, stressful events such as the outbreaks of public health emergencies have been linked to increases in IPV, possibly due to the added stress, anger, frustration and isolation in crisis situations (Bowles, 2020; Smyth *et al.*, 2021). Stress theory postulates that IPV is a way for perpetrators to release their stress and heightened pressure during crises often leads individuals to commit IPV (Farrington, 1986). For example, violence against women and girls increased when the Ebola virus epidemic hit West Africa in 2014–2016 (Usta *et al.*, 2021; Yasmin, 2016). In the recent coronavirus outbreak (COVID-19), fear of COVID infection, stay-at-home policies and job loss/unemployment have increased household stress worldwide (Barrett, 2020; Li *et al.*, 2023; Su *et al.*, 2022; U.S. Bureau of Labor Statistics, 2020), triggering interpersonal conflict at home and potentially IPV. In China, the police department of Jianli County in Hubei Province received 162 domestic violence complaints in January 2020 – more than three times higher than the 47 cases reported during the same month in the previous year (Zhang, 2020). In the U.S., the number of IPV survivors reaching out increased during COVID-19 (Lee, 2020) and in a study (Peitzmeier *et al.*, 2021) where the results did not reveal an increase in the overall prevalence of IPV, the IPV severity increased and novel cases of IPV occurred in relationships that had not been abusive prior to COVID.

It is worth noting that not all data indicate an increase in IPV during COVID-19. For instance, the New South Wales crime statistics suggested no change in domestic violence figures from March 2019 to March 2020 (Freeman, 2020). Data from the International Sexual Health and Reproductive health study which comprises 15,336 participants in 30 countries even showed somewhat reduced IPV during COVID-19 in 2020 (Campbell *et al.*, 2021). However, these results should be interpreted with caution, as researchers in the field have warned that rates of IPV probably did not decrease during the pandemic, but rather that IPV survivors were unable to report IPV victimization safely while being confined to homes together with abusers (Evans *et al.*, 2020; Peitzmeier *et al.*, 2021).

In addition to concerns about a global surge in IPV during public health crises, advocates especially expressed fears of exacerbated vulnerability to IPV for women (Evans *et al.*, 2020; Guterres, 2020; Peitzmeier *et al.*, 2021). Admittedly, IPV research (mostly based on data in Western and more developed economies) has revealed that both men and women could act as perpetrators or victims (Archer, 2006; Hamberger and Larsen, 2015; Straus, 2011), but across the world, a higher lifetime prevalence of experiencing IPV has been reported for women, who also tend to be overrepresented in terms of severe injuries and deaths resulting from IPV (World Health Organization, 2010). In mainland China, the lifetime prevalence of IPV victimization for women is three times the estimate for men (Hu *et al.*, 2021). In the COVID-19 era, however, whether the pandemic has disproportionately impacted women in experiencing IPV remains open to empirical examination, and if so, what risk factors may be associated with the increases in IPV for men and women need to be investigated.

To address these key research questions, we conducted an online cross-sectional survey study during the 2022 Shanghai lockdown. In March 2022, the second wave of the pandemic broke out in Shanghai, prompting the government to implement a city-wide lockdown, requiring all residents to stay at home to control the spread of the virus (Hall *et al.*, 2023). The societal shutdown resulted in many individuals losing their sources of income, causing significant economic pressure which likely increased the risk of IPV victimization for both men and women (Barrett, 2020; Li *et al.*, 2023). Additionally, stay-at-home orders might have lead to additional stressors that could trigger more conflicts at home and potential IPV (Evans *et al.*, 2020; Guterres, 2020; Peitzmeier *et al.*, 2021). Regarding gender (a)symmetry in experiencing IPV and being adversely impacted by the COVID-related lockdown, although the nationwide data suggested a higher lifetime prevalence for women (Hu *et al.*, 2021), this might not apply to Shanghai (one of the most economically and socially developed cities in China) where there are even 5% more women with college degrees or higher levels of education in the workforce than men (Lu, 2015).

Therefore, this study aims to examine the prevalence and various types of IPV and the associated risk factors for men and women during the 2022 Shanghai lockdown, as well as to explore the gender (a)symmetry of IPV victimization in this population. Earlier studies on IPV during COVID-19 mainly based their estimates of IPV on suboptimal proxy measures such as official crime or helpline data (e.g., Ceroni *et al.*, 2023; Freeman, 2020; Graham-Harrison, 2020), which could have led to underestimated IPV rates due to the limited reporting channels during lockdowns. In the present study, we use the more direct self-report survey approach to collect these data. To our knowledge, this is the very first study that directly examined the prevalence, severity or types, and correlates of IPV for men and women during the 2022 Shanghai lockdown.

## Methods

### Participants

**Figure 1.**
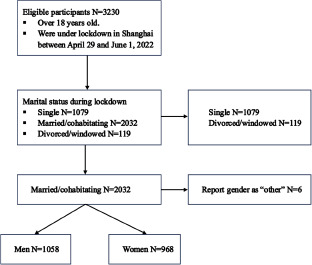
Flow diagram for study participants.

Data were drawn from a cross-sectional online survey conducted in Shanghai between April 29 and 1 June 2022 (from the middle to the end of the lockdown period). A total of 3230 participants were recruited via purposive sampling to reach a geographic target sample of 200 residents in each of the 16 Shanghai districts (Hall *et al.*, 2023). Participants (1058 men and 968 women) who self-reported being married or cohabitating during the lockdown were included in our data analysis. We developed the survey on Wenjuanxing, a Chinese online questionnaire platform. The IP address function of Wenjuanxing locates participants’ network addresses automatically. These addresses were used to confirm that participants were in Shanghai at the time of the survey. Digital informed consent was obtained before study perception. Each participant who completed the survey was offered 6 Chinese yuan (∼$1 USD) (see the process of participant selection in Figure 1). The study was approved by the NYU Shanghai Institutional Review Board.

### Measures and instruments

#### Lockdown-related stressors

Key economic and household stressors were assessed. First, we assessed financial stressors caused by the lockdown, including income loss (yes/no), job loss (yes/no), financial worries (not at all, not more than usual, more than usual and much more than usual) and job worry (yes/no). We assessed household burden, including the number of children home during the lockdown, and whether the family was prepared to undergo the lockdown (‘At the beginning of the lockdown, did your family have sufficient stock of the following items (food, water and daily supplies) for at least 1 week?’ (yes/no)).

#### Intimate partner violence

Participants completed an adapted version of the Extended-Hurt, Insult, Threaten, Scream (E-HITS) screening tool to assess IPV exposure. The E-HITS has demonstrated reliability and validity in assessing IPV in healthcare settings (Chan *et al.*, 2010; Goldstein *et al.*, 2023). Participants were asked, ‘How often did a partner (1) physically hurt you? (2) insult you or talk down to you? (3) threaten you with harm? (4) scream or curse at you? and (5) force you to have sexual activities?’ during lockdown. Responses were scored on a 5-point Likert scale (1 = never to 5 = frequently). Total scores were calculated from the sum of item ratings (range, 5–25), with a score of ≥7 defined as IPV exposure. Following Relyea *et al*. (2020), any answer of two (rarely) or higher on any item was coded as experienced that subtype of IPV.

#### Study covariates

Study covariates included age group (women: 18–24, 25–34, 35–44, ≥45; men: 18–24, 25–34, 35–44, 45–54, ≥55), educational attainment (less than high school, high school, college graduate or more), household income in Chinese Yuan (<4000, 4001–8000, 8001–15,000, 15,001–30,000, 30,000 or higher), employment status (employed, part-time, unemployed), previous psychiatric diagnosis by a mental health professional (yes/no) and migration status. In China, the *hukou* is a household registration that is bound to a particular rural or urban location. People with a Shanghai hukou can enjoy preferential policies, like local healthcare and educational resources in the city. There are two ways to obtain a Shanghai hukou, being born in Shanghai or migrating to the city and meeting certain occupational or other criteria. Four kinds of migration status were investigated in this study: Shanghai local, migrant with hukou, migrant without hukou and temporary migrants who do not intend to permanently migrant to Shanghai.

### Statistical analysis

All analyses were weighted to adjust for deviations between the sample and the most recent Shanghai census. Weights were calculated by utilizing logistic regression models to create an inverse probability of sampling weights to account for the differences in the distribution of covariates (i.e., district and age) between the study population and the 2020 Shanghai Census data (Cole and Stuart, 2010; Hall *et al.*, 2023).

We estimated the prevalence of IPV and subtypes of IPV. Study covariates were described using raw frequencies and weighted percentages. Firstly, we used two-tailed χ^2^ tests to estimate the bivariable associations between demographic characteristics, lockdown-related stressors and IPV victimization. Then, bivariable logistic regression analyses were conducted to explore the unadjusted direct associations between demographic characteristics, lockdown-related economic and household stressors and exposure to IPV for men and women, respectively. Multivariable logistic regression analyses then adjusted for demographic characteristics (age, income, education, migrant status, past psychological diagnosis), regressing lockdown factors on IPV and IPV subtypes in separate analyses. Analyses were conducted using *svy* commands in Stata/MP 17.0, with statistical significance at *p* < 0.05. The 95% confidence intervals (CIs) for the prevalence and odds ratios were calculated.

## Results

Most men were aged 25–44 years (715/48.8%) (*median age*: 38, *IQR*: 33–47, range: 19–88), and the majority of women were 25–34 years old (525/49.4%) (*median age*: 34, *IQR*: 30–39.5, range: 19–86). Most men (621/56.1%) and women (610/66.7%) reported a university degree, with a family income between 8000 and 15,000 Chinese yuan (men: 361/30.0%; women: 267/29.6%), and most of them were employed (men: 887/87.6%; women: 792/71.7%). Nearly half the sample of men (587/42.1%) and women (533/49.7%) reported being migrants without a Shanghai household registration (i.e., *hukou*). Twenty-nine (1.4%) men and forty-eight (20.6%) women reported a previous psychiatric diagnosis (see Table 1).

**Table 1.** Participant Characteristics

	N (%)	
	Men	Women
**Overall**	1058 (100)	968 (100)
**Age**		
18−24	20 (1.1)	27 (2.6)
25−34	337 (23.2)	525 (49.4)
35−44	378 (25.6)	264 (24.6)
45−54/≥45	203 (14.6)	152 (23.4)
≥55	120 (35.5)	–
**Education**		
Less than high school	173 (18.1)	162 (14.1)
High school	264 (25.8)	196 (19.2)
College graduate or higher	621 (56.1)	610 (66.7)
**Income**		
Less than 4000	59 (3.6)	90 (8.0)
4001−8000	274 (33.0)	231 (25.7)
8001−15000	361 (37.0)	267 (29.6)
15,001−30000	261 (19.0)	262 (25.1)
30000 or higher	103 (7.4)	118 (11.6)
**Employment**		
Employed	887 (87.6)	792 (71.7)
Part–time	64 (4.1)	48 (5.2)
Unemployed	107 (8.3)	128 (11.7)
**Migrant status**		
Shanghai local	340 (48.0)	275 (34.1)
Migrant with hukou	140 (9.9)	160 (16.2)
Migrant without hukou	344 (24.6)	291 (25.7)
Temporary	234 (17.5)	242 (24.0)
**Past psychological diagnosis**		
No	1029 (98.6)	920 (79.4)
Yes	29 (1.4)	48 (20.6)

**Figure 2.**
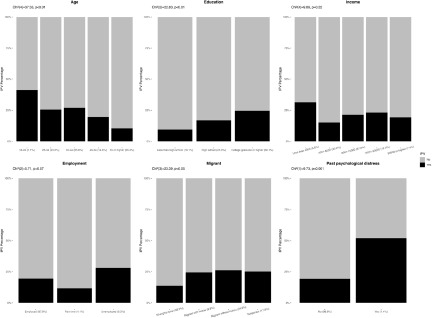
Associations between sociodemographic characteristics and intimate partner violence for men (*N* = 1058). Bar charts represent the proportion of IPV victimization by sociodemographic characteristics. All percentages were weighted. *P* values were calculated using the two-sided Pearson’s chi-squared test.

The prevalence of IPV experienced by men was significantly different by age, education, migrant status, past psychological diagnosis, income loss, job loss and finance worry. For women, they differed by migrant status, past psychological diagnosis, income loss, job loss, finance worry and food preparation (see Figure 2–Figure 5).

**Figure 3.**
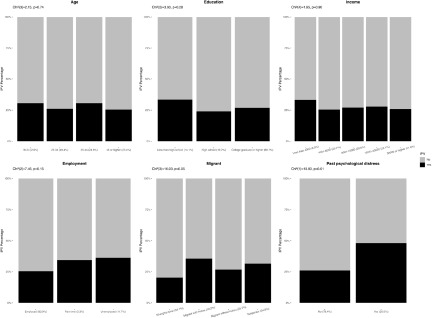
Associations between sociodemographic characteristics and intimate partner violence for women (*N* = 968). Bar charts represent the proportion of IPV victimization by sociodemographic characteristics. All percentages were weighted. *P* values were calculated using the two-sided Pearson’s chi-squared test.

**Figure 4.**
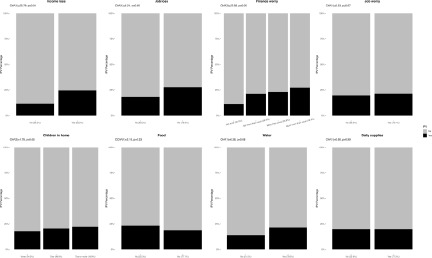
Associations between lockdown-related stressors and intimate partner violence for men (*N* = 1058). Bar charts represent the proportion of IPV victimization by lockdown-related stressors. All percentages were weighted. *P* values were calculated using the two-sided Pearson’s chi-squared test.

**Figure 5.**
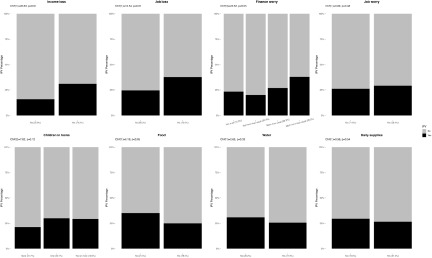
Associations between lockdown-related stressors and intimate partner violence for women (*N* = 968). Bar charts represent the proportion of IPV victimization by lockdown-related stressors. All percentages were weighted. *P* values were calculated using the two-sided Pearson’s chi-squared test.

Nearly, 20% (19.8%, 95% CI: 16.1–24.0) men and 30% (27.1%, 95% CI: 23.1–31.4) women experienced IPV during lockdown. Verbal violence was the most prevalent form of IPV for both men and women (insulted you: women: 29.2%, 95% CI: 25.8–34.4; men: 22.3%, 95% CI: 18.4–26.8; screamed or cursed at you: women: 30.6%, 95% CI: 26.3–35.2; men: 23.8%, 95% CI: 19.8–28.4) (see Table 2).

**Table 2.** Prevalence of IPV for men (*n* = 1058) and women (*n* = 968)

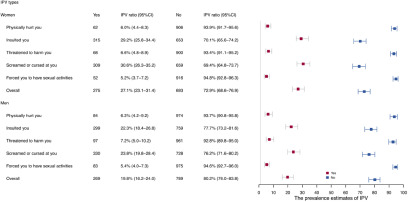

*Notes*: Overall means the prevalence of IPV experienced by men and women calculated by the screening threshold score of E-HITS. IPV types means the prevalence of IPV types experienced by men and women calculated by the screening threshold score of each type.

### Correlates of overall IPV by sex

Table 3 reports the results of unadjusted binary logistic regression analysis. The multivariable analyses suggested several correlates of IPV after adjusting for demographic characteristics (see Table 4). For economic and household stress, women who experienced income loss (adjusted OR [aOR] = 2.42, 95% CI: 1.28–4.56), job loss (aOR = 1.73, 95% CI: 1.02–2.92) and worried about finances much more than usual (aOR = 1.89, 95% CI: 1.00–3.57) compared to not experiencing these losses during lockdown had increased odds of IPV. Women who had one child at home (aOR = 1.81, 95% CI: 1.12–2.92) compared to no child also experienced increased odds of IPV. Women who did not have enough food for the family (aOR = 1.67, 95% CI 1.04-2.70) compared to the group with sufficient preparation were associated with increased odds of IPV. In contrast, the experience of IPV for men was not associated with any of these factors.

**Table 3.** Bivariable relationships between lockdown-related stressors and intimate partner violence by type of experienced IPV

	Intimate partner violence		Physically hurt you		Insulted you		Threatened to harm you		Screamed or cursed at you		Forced you to have sexual activities	
	OR (95%CI) Men	OR (95%CI) Women	OR (95%CI) Men	OR (95%CI) Women	OR (95%CI) Men	OR (95%CI) Women	OR (95%CI) Men	OR (95%CI) Women	OR (95%CI) Men	OR (95%CI) Women	OR (95%CI) Men	OR (95%CI) Women
*Income loss due to lockdown*												
No	1	1	1	1	1	1	1	1	1	1	1	1
Yes	**2.48** **(1.29−4.75)**	**2.35 (1.26−4.37)**	**3.18 (1.00−10.10)**	2.46 (0.83−7.32)	**2.90 (1.53−5.53)**	**2.30 (1.25−4.20)**	**3.17 (1.10−9.12)**	**3.94 (1.75−8.89)**	**2.33 (1.29−4.23)**	1.68 (0.89−3.16)	1.98 (0.75−5.28)	2.35 (0.81−6.81)
*Job loss due to lockdown*												
No	1	1	1	1	1	1	1	1	1	1	1	1
Yes	**1.71 (1.11−2.64)**	**1.86 (1.22−2.86)**	**2.05 (1.02−4.15)**	1.56 (0.79−3.11)	**1.57 (1.02−2.41)**	**1.91 (1.26−2.90)**	**2.09 (1.12−3.91)**	1.10 (0.54−2.26)	**1.53 (1.01−2.32)**	**1.85 (1.21−2.81)**	1.71 (0.93−3.15)	1.89 (0.91−3.88)
*Finance worry*												
Not at all	1	1	1	1	1	1	1	1	1	1	1	1
Not more than usual	2.11 (0.88−5.08)	0.81 (0.39−1.70)	1.19 (0.27−5.31)	0.39 (0.11−1.37)	1.54 (0.67−3.52)	0.63 (0.31−1.30)	1.57 (0.35−7.00)	0.55 (0.18−1.62)	**2.45 (1.10−5.49)**	0.60 (0.29−1.26)	2.48 (0.91−6.74)	0.44 (0.11−1.67)
More than usual	2.30 (1.04−5.36)	1.20 (0.66−2.19)	1.40 (0.35−5.69)	0.52 (0.18−1.55)	**2.60 (1.19−5.66)**	1.30 (0.74−2.31)	1.71 (0.41−7.06)	0.88 (0.33−2.33)	**2.33 (1.11−4.93)**	1.02 (0.56−1.85)	**2.55 (1.10−5.94)**	1.09 (0.34−3.48)
Much more than usual	**2.94 (1.33−6.49)**	**1.99 (1.11−3.57)**	1.40 (0.36−5.47)	1.25 (0.46−3.40)	**2.44 (1.16−5.15)**	**2.02 (1.15−3.54)**	1.83 (0.46−7.36)	1.13 (0.45−2.87)	**2.59 (1.26−5.34)**	1.62 (0.90−2.90)	**3.42 (1.56−7.54)**	0.99 (0.29−3.36)
*Job worry*												
No	1	1	1	1	1	1	1	1	1	1	1	1
Yes	1.12 (0.67−1.87)	1.16 (0.77−1.75)	1.64 (0.77−3.53)	0.90 (0.43−1.89)	1.13 (0.67−1.88)	1.29 (0.86−1.91)	1.17 (0.57−2.42)	1.56 (0.80−3.06)	0.89 (0.54−1.48)	1.17 (0.78−1.75)	**2.15 (1.11−4.17)**	0.85 (0.37−1.94)
*Children at home*												
None	1	1	1	1	1	1	1	1	1	1	1	1
One	1.19 (0.67−2.11)	1.58 (0.97−2.57)	1.62 (0.58−4.56)	0.78 (0.36−1.68)	1.31 (0.76−2.27)	1.56 (0.96−2.51)	1.24 (0.50−3.04)	0.88 (0.41−1.86)	1.21 (0.70−2.11)	1.56 (0.96−2.54)	0.87 (0.40−1.90)	1.20 (0.54−2.66)
Two or more	1.32 (0.74−2.39)	1.52 (0.86−2.68)	2.21 (0.87−5.59)	0.92 (0.36−2.37)	1.67 (0.91−3.04)	**1.77 (1.01−3.08)**	1.47 (0.65−3.30)	1.33 (0.52−3.40)	1.02 (0.57−1.82)	1.31 (0.75−2.31)	1.20 (0.49−2.93)	1.14 (0.39−3.33)
*Food*												
*Yes*	1	1	1	1	1	1	1	1	1	1	1	1
*No*	1.29 (0.76−2.22)	**1.61 (1.03−1.61)**	1.49 (0.69−3.22)	0.96 (0.98−3.85)	1.45 (0.87−2.44)	1.54 (0.99−2.38)	1.35 (0.67−2.70)	1.23 (0.60−2.56)	0.93 (0.55−1.56)	1.39 (0.89−2.17)	1.10 (0.57−2.13)	1.72 (0.80−3.70)
*Water*												
*Yes*	1	1	1	1	1	1	1	1	1	1	1	1
No	0.59 (0.33−1.06)	1.30 (0.77−2.17)	0.51 (0.24−1.08)	**3.57 (1.79−7.14)**	0.73 (0.40−1.32)	1.12 (0.68−1.89)	0.56 (0.27−1.14)	**2.04 (1.01−4.17)**	0.71 (0.40−1.27)	1.15 (0.68−1.92)	0.76 (0.38−0.54)	1.45 (0.65−3.23)
*Daily supplies*												
*Yes*	1	1	1	1	1		1	**1**	1	1	1	1
*No*	1.00 (0.56−1.79)	1.15 (0.75−1.75)	0.62 (0.29−1.32)	**2.94 (1.49−5.88)**	1.02 (0.56−1.89)	1.43 (0.94−2.17)	0.57 (0.28−1.18)	2.00 (0.98−4.00)	1.11 (0.63−2.00)	1.12 (0.74−1.72)	0.64 (0.31−1.32)	**2.56 (1.27−5.26)**

*Notes*: OR = odds ratio which is the unadjusted association between the exposure and outcome. Bold estimates are *p* < .05.

### Correlates of IPV types by sex

Women who experienced income loss (insult: aOR = 2.18, 95% CI: 1.19–3.98), job stress (insult: aOR = 1.73, 95% CI: 1.06–2.96; scream: aOR = 1.95, 95% CI: 1.17–3.25) or had at least one child at home (insult: one child: aOR = 1.74, 95% CI: 1.10–2.74; ≥two or more children: aOR = 1.80, 95% CI: 1.05–3.07; scream: one child: aOR = 1.64, 95% CI: 1.03–2.62) compared to their reference groups experienced increased odds of verbal violence (i.e., insult or scream). Women who reported not having enough water for the family experienced increased odds of physical IPV (aOR = 3.33, 95% CI: 1.79–6.25) and threat (aOR = 2.08, 95% CI: 1.04–4.00). Similarly, women who did not have enough daily supplies for family had an associated increased odds of physical IPV (aOR = 2.77, 95% CI: 1.18–4.35) and sexual IPV (aOR = 2.17, 95% CI: 1.03–4.55). Men who lost their jobs experienced an increased odds of physical IPV (aOR = 2.27, 95% CI: 1.09–4.69) and threat (aOR = 2.50, 95% CI: 1.25–5.01). Men who had two or more children (aOR = 2.95, 95% CI: 1.33–7.69) had an increased odds of physical IPV (see Table 4).

**Table 4.** Multivariable relationships between lockdown-related stressors and intimate partner violence by type of experienced IPV

	Intimate partner violence		Physically hurt you		Insulted you		Threatened to harm you		Screamed or cursed at you		Forced you to have sexual activities	
	aOR (95%CI) Men	aOR (95%CI) Women	aOR (95%CI) Men	aOR (95%CI) Women	aOR (95%CI) Men	aOR (95%CI) Women	aOR (95%CI) Men	aOR (95%CI) Women	aOR (95%CI) Men	aOR (95%CI) Women	aOR (95%CI) Men	aOR (95%CI) Women
*Income loss due to lockdown*												
No	1	1	1	1	1	1	1	1	1	1	1	1
Yes	1.76 (0.89−3.51)	**2.42 (1.28−4.56)**	2.86 (0.81−10.05)	1.91 (0.71−5.15)	2.03 (0.99−4.12)	**2.18 (1.19−3.98)**	2.75 (0.83−9.04)	**2.75 (1.14−6.65)**	1.63 (0.87−3.05)	1.53 (0.85−2.75)	1.21 (0.56−2.59)	1.62 (0.54−4.92)
*Job loss due to lockdown*												
No	1	1	1	1	1	1	1	1	1	1	1	1
Yes	1.58 (0.99−2.54)	**1.73 (1.02−2.92)**	**2.27 (1.09−4.69)**	1.12 (0.49−2.58)	1.20 (0.68−2.12)	**1.73 (1.06−2.96)**	**2.50 (1.25−5.01)**	0.75 (0.35−1.60)	1.45 (0.92−2.26)	**1.95 (1.17−3.25)**	1.41 (0.70−2.85)	**2.66 (1.16−6.12)**
*Finance worry*												
Not at all	1	1	1	1	1	1	1	1	1	1	1	1
Not more than usual	1.65 (0.72−3.81)	0.83 (0.41−1.68)	0.97 (0.28−3.34)	0.40 (0.11−1.46)	1.16 (0.53−2.53)	0.65 (0.33−1.29)	1.21 (0.36−4.09)	0.59 (0.20−1.74)	1.96 (0.94−4.11)	0.61 (0.30−1.21)	2.05 (0.70−6.00)	0.51 (0.14−1.82)
More than usual	1.63 (0.77−3.45)	1.16 (0.63−2.16)	1.03 (0.34−3.16)	0.48 (0.15−1.30)	1.70 (0.84−3.48)	1.25 (0.69−2.25)	1.24 (0.40−3.83)	0.61 (0.23−1.67)	1.58 (0.82−3.05)	0.96 (0.53−1.75)	1.86 (0.73−4.75)	0.86 (0.28−2.68)
Much more than usual	1.86 (0.85−4.07)	**1.89 (1.00−3.57)**	0.89 (0.30−2.67)	0.87 (0.29−2.60)	1.41 (0.68−2.92)	**1.94 (1.05−3.59)**	1.25 (0.41−3.80)	0.68 (0.25−1.87)	1.67 (0.83−3.35)	1.63 (0.89−2.98)	2.40 (0.92−6.26)	0.77 (0.22−2.68)
*Job worry*												
No	1	1	1	1	1	1	1	1	1	1	1	1
Yes	0.79 (0.49−1.27)	1.21 (0.81−1.80)	1.33 (0.60−2.94)	0.84 (0.38−1.84)	0.80 (0.50−1.29)	1.31 (0.89−1.92)	0.87 (0.40−1.56)	1.28 (0.62−2.61)	**0.61 (0.39−0.93)**	1.16 (0.79−1.72)	1.83 (0.94−3.59)	0.63 (0.26−1.51)
*Children at home*												
None	1	1	1	1	1	1	1	1	1	1	1	1
One	1.30 (0.71−2.38)	**1.81 (1.12−2.92)**	2.01 (0.66−6.12)	0.91 (0.44−1.90)	1.45 (0.83−2.54)	**1.74 (1.10−2.74)**	1.45 (0.53−3.97)	1.02 (0.48−2.17)	1.22 (0.71−2.11)	**1.64 (1.03−2.62)**	0.75 (0.34−1.62)	1.38 (0.56−3.37)
Two or more	1.38 (0.79−2.40)	1.58 (0.91−2.75)	**2.95 (1.33−7.69)**	0.98 (0.38−2.55)	1.65 (0.94−2.91)	**1.80 (1.05−3.07)**	1.76 (0.75−4.13)	1.44 (0.59−3.50)	0.96 (0.57−1.63)	1.28 (0.75−2.17)	1.07 (0.47−2.46)	1.13 (0.37−3.50)
*Food*												
*Yes*	1	1	1	1	1	1	1	1	1	1	1	1
*No*	1.01 (0.60−1.72)	**1.67 (1.04−2.70)**	1.19 (0.57−2.50)	1.92 (0.92−4.00)	1.09 (0.65−1.82)	**1.59 (1.02−2.44)**	1.06 (0.53−2.13)	1.20 (0.54−2.63)	0.71 (0.54−1.19)	1.45 (0.93−2.27)	0.89 (0.47−1.69)	1.59 (0.72−3.45)
*Water*												
*Yes*	1	1	1	1	1	1	1	1	1	1	1	1
*No*	0.56 (0.31−1.03)	1.28 (0.81−2.04)	**0.47 (0.22−0.98)**	**3.33 (1.79−6.25)**	0.68 (0.38−1.23)	1.12 (0.72−1.75)	0.49 (0.24−1.01)	**2.08 (1.04−4.00)**	0.72 (0.40−1.28)	1.18 (0.75−1.85)	0.70 (0.34−1.47)	1.69 (0.79−3057)
*Daily supplies*												
*Yes*	1	1	**1**	**1**	1		1	**1**	1	1	1	1
No	0.99 (0.58−1.69)	0.98 (0.65−1.49)	0.54 (0.26−1.12)	**2.27 (1.18−4.35)**	0.90 (0.52−1.54)	1.22 (0.81−1.82)	0.50 (0.24−1.01)	1.35 (0.68−2.70)	1.19 (0.70−2.04)	1.01 (0.67−1.52)	0.56 (0.26−1.20)	**2.17 (1.03−4.55)**

*Notes*: aOR = adjusted odds ratio. Models were adjusted for demographic characteristics (age, income, education, employment, migrant status, past psychological distress). Bold estimates are *p* < .05.

## Discussion

The current study utilized survey data from a large sample that experienced the Shanghai 2022 lockdown and analysed IPV prevalence and corresponding risk factors stratified by sex. Results revealed a gendered pattern whereby significant associations between economic/household stress and IPV were largely experienced by women but with few effects only for physical violence for men.

Compared to men, women were more likely to experience IPV during the lockdown (27.1% women vs. 19.8% men). This is near the prevalence of lifetime IPV in China for women but more than twice that previously reported for men (Yang *et al.*, 2019). The prevalence of verbal violence was higher than other IPV types for both men and women. Although there is limited comparable data in Shanghai exploring the prevalence of IPV, a study conducted in 2013 used data from 216 male and female adolescents, aged 15–19, who were immigrants to explore the prevalence and health impact of IPV, and the result showed the past-year IPV prevalence was 10.2% in Shanghai (Decker *et al.*, 2014). Another study conducted among married rural migrant women of reproductive age in April and May of 2010 in Shanghai found that 18.7% women reported any kind of IPV (emotional, physical or sexual abuse) in the past year. Overall, the prevalence of IPV was higher than estimates obtained before COVID-19.

Over 60% of men and 70% of women experienced income loss and over 10% of men and women lost their jobs during the lockdown. Income loss, unemployment and financial worry were all correlates of experiencing increased overall IPV among women but not men. According to Connell’s (1987) integrative theory of gender and power (TGP), people with weaker division of labour are more likely to become victims of IPV (Connell, 1987). Although more women work now, unemployment was a key correlate of their experienced IPV. Meanwhile, considering the higher percentage income loss among men, violence may have played a compensatory role. The traditional gender identity of men – being responsible for financial stability – may have led to instability of gender power structure during lockdown. According to TGP, the increase in IPV victimization may have been a tool used to compensate for income loss or unemployment and to assert dominance through violent means in the current study (Clare *et al.*, 2021). Based on our results, women should not only fulfil the obligations of the household but may have also be blamed for their finance loss.

The results showed that men also reported IPV, especially those with higher education, which was supported by Zhang *et al*. (2015). One possible explanation is that at least some portion of men’s reporting of experienced IPV may be the violence women engage in for self-defence (Kimmel, 2002; Zhang *et al.*, 2015). Women were likely to use violence to protect themselves or retaliate against prior violence (Allen, 2011). Besides, the focus of IPV measurement in this study was the violence itself, which fails to reflect to reasons of violence and behaviours leading to violence. The measurements without the context of violence may exaggerate the frequency of men self-reporting victimization (Zhang *et al.*, 2015). Future research should incorporate additional information to clarify the context of violence against men. In fact, the results indicated that increased violence was reported by men who lost their job, had two more children at home and did not have sufficient water for their family. Women may have perpetrated verbal and physical IPV when their partners were unable to fulfil their social role as a breadwinner (Oinas, 2018). Although IPV reported by women was more severe, victimization of men should not be ignored.

Migration status was a key risk factor for both men and women, compared to Shanghai locals, migrants experienced a higher burden of IPV, which is supported by previous studies conducted in China (Chen *et al.*, 2016; Li and Wang, 2023; Tu and Lou, 2017). However, empirical work surrounding migrants and IPV has mostly focused on women (Chen *et al.*, 2016). In fact, both men and women migrants may face employment, income and social welfare insecurity (Wong *et al.*, 2008). Income and job instability among migrants may increase the possibility of IPV.

### Limitations

There are several limitations that should be noted in this study. First, limited by the lockdown, our data were collected by online self-report questionnaires. Self-report data may be influenced by social desirability and recall biases, which might lead to misestimation of the prevalence of IPV. However, given the anonymity and confidentiality protections in the study, the participants might not have been motivated to intentionally give socially desirable responses. Second, the research design of this study was cross-sectional, and conclusions cannot be drawn regarding causality. Third, the participants were in romantic relationships but may not have been paired. The TGP emphasizes gender actions and relations in civil institutions. Data from paired couples living together may be more helpful to understand the relationship between labour, power and IPV. Finally, the data collection and assumptions of romantic partnerships adhere to a heteronormative narrative, and the lived experiences of LGBTQ+ participants are not reflected in these data. Future studies should examine IPV from an intersectional lens, exploring gender, sexuality and violence within a Chinese context. Finally, it should be noted that in this study all participants came from Shanghai, arguably the most developed city in China. Compared to other provinces, Shanghai has a close contact with Western culture, more developed community-based organization sectors and convenient welfare to residents. Our results should be interpreted with caution when generalized to other populations in China. Future work is also needed to understand the nature of IPV in Shanghai and among men, outside the COVID-19 context as current data are limited.

Notwithstanding these limitations, this study provided key insights and important practical implications. First, income loss, unemployment and household burden were all risk factors for overall IPV experienced by women, but the same pattern was not observed for men, suggesting a key gendered response to threats against masculine roles still impacted the risk of IPV against women in Shanghai (Clare, 2021). The economic function of being a breadwinner was limited by a public health emergency, which may lead to IPV as a compensatory mechanism for men to balance their predominance in the gender and power structure. The Women’s Federation or other social welfare institutions should be strengthened to maintain access to provide help for women suffering IPV during public health emergencies. Second, men are also at risk of IPV, especially those with higher levels of education, experiencing job loss due to lockdown, having two or more children at home or not having sufficient water for family. Future research should examine the risk factors and motivation of IPV against men to gain further understanding of gender (a)symmetry of IPV. Finally, verbal IPV appears to be of particularly high prevalence during the Shanghai 2022 lockdown. Interventions to promote healthy conflict resolution skills within intimate partnerships may be beneficial to reduce this from of IPV in the future.

## Conclusion

During the Shanghai 2022 lockdown, 27.1% women and 19.8% men met screening thresholds for total IPV. This is greater than the prevalence of lifetime IPV experienced by women (25%) and men (8%) in mainland China (Hu *et al.*, 2021). The occurrence of verbal violence was higher than other kinds of IPV. The gender pattern of IPV experienced by men and women is different. Economic stress and household burden were both risk factors for overall IPV and all IPV types against women. While for men, economic hardship may be the root reason to experience verbal or physical IPV. These findings highlighted the importance of improving gender equality awareness and interventions in China and evidence for the surplus of IPV experienced during the lockdown period.
